# The Use of Auricular Cartilage Grafts in Septorhinoplasty: A Dual-Centre Study of Donor Site Patient-Reported Outcome Measures

**DOI:** 10.7759/cureus.26547

**Published:** 2022-07-04

**Authors:** Ravi Kumar, Adnan Darr, Charn Gill, Navdeep Bhamra, Nina Mistry, James Barraclough

**Affiliations:** 1 Otolaryngology, The Royal Wolverhampton NHS Trust, Wolverhampton, GBR

**Keywords:** reconstruction, grafts, auricular, cartilage, septorhinoplasty, rhinoplasty

## Abstract

Objectives

The use of autologous grafts is a key aspect of contemporary septorhinoplasty. When septal cartilage is deficient, auricular cartilage serves as a biocompatible, readily accessible alternative. Our study aimed to assess donor site patient-reported outcome measures (PROMs) where auricular cartilage has been harvested for use in septorhinoplasty, adding to the limited existing literature on this topic.

Design

A dual-centre, single-surgeon retrospective analysis of patients undergoing septorhinoplasty surgery with augmentation using auricular cartilage grafts was conducted. Grafts were harvested using an anterior anti-helical approach. Patients were followed up at one week, three months and 12 months post-operatively. Donor site outcomes were assessed across several physical and psychological domains by adapting the EAR-Q questionnaire, which was administered via telephone consultation. Responses were quantified using a Likert scale.

Results

A total of 22 patients met our inclusion criteria. Four were lost to follow-up, five were non-responders and one case was excluded due to documentation of body dysmorphic disorder. A significant proportion of patients reported no reduction in quality of life (QOL) or confidence attributed to donor site cosmesis. High satisfaction was noted with anti-helical donor site scars. Although noticeable differences in shape and symmetry were reported, these had negligible effects across psychological domains.

Conclusions

Preliminary results suggest high levels of patient satisfaction, with minimal physical and psychological donor site sequelae following auricular cartilage harvest in septorhinoplasty. Subsequent studies should involve the use of validated questionnaires, coupled with larger patient cohorts in order to provide further data for statistical analysis.

## Introduction

The use of autologous cartilage grafts to achieve satisfactory long-term functional and aesthetic results is a key aspect of contemporary septorhinoplasty [1-3]. Successful reconstruction of the nasal framework can be hindered when septal cartilage is insufficient or of poor structural integrity. Auricular cartilage is an easily accessible source of graft material, which can be harvested and tailored to the target defects or aid augmentation [2,4]. Several disadvantages of this approach have been postulated within the literature, including donor site pain, deformity and subsequent impairment in quality of life (QOL) [5]. Autologous costal cartilage is often cited as a preferable source owing to its abundance, stiffness and relative donor site cosmesis [3]. Optimal graft material remains unclear, with relative complication rates of several methods cited within the literature [6,7]. Ultimately, the decision regarding the source of graft material and approach is a combination of both patient and operator preference.

Important surrogate markers of success in septorhinoplasty surgery are patient perception and satisfaction. To date, there are limited studies that formally assess both physical and psychological patient-reported outcome measures (PROMs) at the donor site where auricular cartilage has been harvested for septorhinoplasty surgery.

## Materials and methods

A single-surgeon, dual-centre retrospective case-note analysis of patients undergoing septorhinoplasty surgery with auricular cartilage grafts between January 2016 and December 2019 was conducted. An anterior approach for graft harvest via incision along the anti-helix is our preferred technique, relating to ease of access, minimal requirement for retraction, and optimal visualisation of the anterior limits of dissection. Patients were followed up at one-week, three-month and 12-month intervals. Exclusion criteria included non-anterior/anti-helical approaches to graft harvesting, revision/multiple harvesting attempts at the same site, and a follow-up period of fewer than 12 months. Donor site outcomes were assessed across several physical and psychological domains by identifying and adapting key items defined within the EAR-Q questionnaire developed by Klassen et al. [8]. Semi-structured interviews of 20 randomly selected rhinology patients were conducted by two independent investigators in an outpatient clinic setting to establish domains for data collection.

Physical domains included donor site pain, numbness, sensitivity, hearing impairment, scarring, as well as perceived alteration in size, shape and symmetry. Psychological domains included the effect of donor site appearance on level of confidence, self-consciousness and QOL. A four-point Likert scale (score: 0, 1, 2, 3) was utilised to quantify responses within each domain. Data were collated over telephone by two independent investigators located at each hospital site using a standardised questionnaire.

## Results

A total of 22 patients were identified. Of these, four patients were excluded due to insufficient follow-up, five patients were non-respondents and one case was excluded due to documented evidence of body dysmorphic disorder, which we felt would have an adverse skew on the outcome and overall data. Of the 12 patients meeting our inclusion criteria, seven were male and 5 were female, with a mean age of 42.1 years (SD = 13.3). The majority of cases (66.7%, n = 8) requiring use of autologous conchal cartilage were documented as revision cases. The percentage mode of responses per domain is summarised in Tables 1-3.

**Table 1:** Physical domain questions and responses Key: 0 = not at all, 1 = a little bit, 2 = quite a bit and 3 = very much

Since your operation have you experienced any …?						
Outcome measure	Likert score no.				Mode	Mode percentage (n=12)
	0	1	2	3		
Pain	8	1	1	2	0	66%
Numbness	10	1	0	1	0	83%
Sensitivity	9	2	0	1	0	75%
Difficult hearing	10	1	1	0	0	83%

**Table 2:** Psychological domain questions and responses (1) Key: 0 = not at all, 1 = a little bit, 2 = quite a bit and 3 = very much

How troubled are you by the … of your operated ear?						
Outcome measure	Likert score no.				Mode	Mode percentage (n=12)
	0	1	2	3		
Pain	8	1	1	2	0	66%
Numbness	10	1	0	1	0	83%
Sensitivity	9	2	0	1	0	75%
Difficult hearing	10	1	1	0	0	83%

**Table 3:** Psychological domains questions and responses (2) Key: 0 = not at all, 1 = a little bit, 2 = quite a bit and 3 = very much

How much has the appearance of your operated ear negatively impacted your ...?						
Outcome measure	Likert score no.				Mode	Mode percentage (n=12)
	0	1	2	3		
Pain	8	1	1	2	0	66%
Numbness	10	1	0	1	0	83%
Sensitivity	9	2	0	1	0	75%
Difficult hearing	10	1	1	0	0	83%

**Figure 1:**
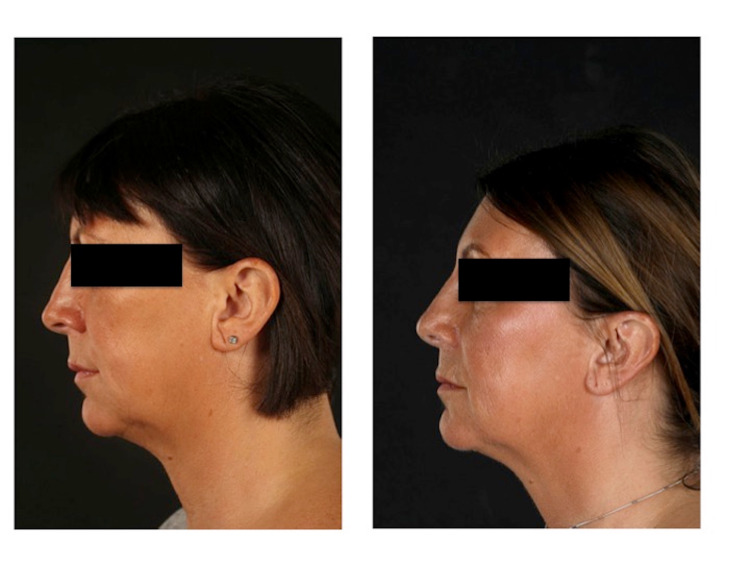
Left donor ear pre-operation vs. post-operation

**Figure 2:**
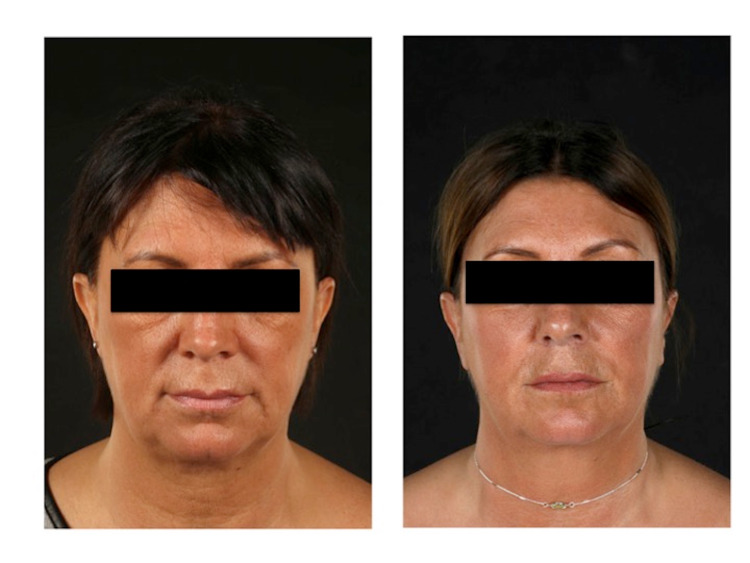
Left donor ear pre-operation vs. post-operation AP symmetry AP: anteroposterior

**Figure 3:**
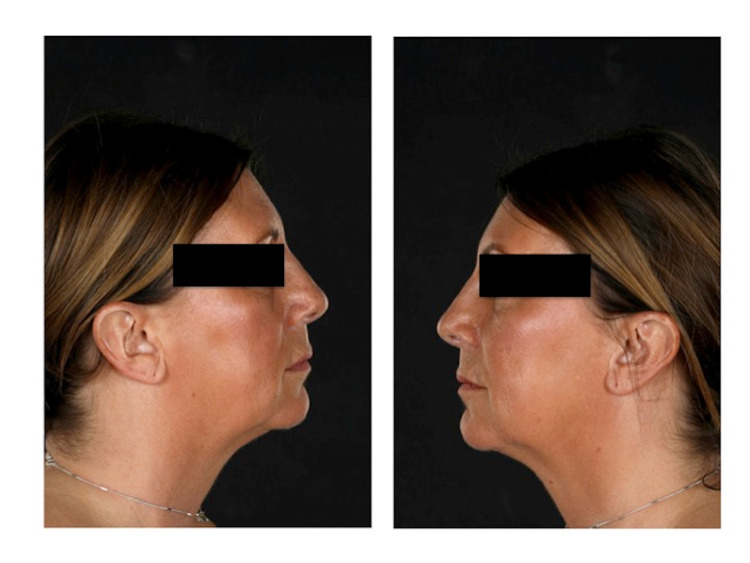
Post-operative symmetry right ear vs. left (donor) ear

The most common response skewed towards Likert scores of 0, suggesting overall high patient satisfaction. Eighty-three percent of patients (n = 10) reported no reduction in QOL from donor site cosmesis. Ninety-two percent (n = 11) were unaffected by the appearance of anti-helical donor site scarring. Variable responses were received when asked about long-term post-operative pain; 66% of patients (n = 8) stated they never had pain, while sixteen percent (n =2) reported constant donor site pain, with the remaining patients falling in between. Eighty-three percent of patients reported no numbness (n = 10) and 75% stated they never suffered donor site sensitivity (n = 9). Eighty-three percent of patients noted no post-operative deficit in hearing (n = 10). Although noticeable differences in shape and symmetry when compared to the contralateral ear were reported, with 58% of patients in each domain (n = 7) responding with scores of one to three, a corresponding drop in satisfaction across psychological domains was not seen in the majority of respondents. Ninety-two percent of patients (n = 11) stated they had no reduction in post-operative confidence due to the appearance of the donor's ear. Pre- and post-operative clinical photographs of one patient are included for reference in Figures 1-3.

## Discussion

Autologous cartilage is a readily available and biocompatible graft material in nasal reconstruction. A review of the literature suggests that the auricle and rib are common donor sites in septorhinoplasty surgery [1,2,9]. Auricular cartilage can be obtained with relative ease, and its proximity to the nose, coupled with surgeon preference makes it the preferred technique at our institutions [10]. The contours of the pinna render this a versatile source of cartilage, which can be tailored to replicate curvilinear nasal anatomy [11]. Costal cartilage is abundant and may be a useful alternative when a large amount of graft material is required [9]. There is a distinct lack of high-level evidence within the literature to support one technique over the other, with relative complication rates of several methods cited within the literature (Table 4) [6,7]. The ultimate aim of septorhinoplasty surgery is to modify the nasal framework to optimise cosmesis and function for the benefit of the patient. In lieu of an evidence-based choice between techniques on the basis of clinical endpoints and complications, PROMs may represent a surrogate benchmark to differentiate between approaches.

**Table 4:** Advantages and disadvantages of donor sites for grafts in septorhinoplasty procedures The table is adapted from Bussi et al. [6] and Justicz et al. [7].

	Advantages	Disadvantages
Septal cartilage (autologous)	Easily accessible; no additional donor site morbidity; minimal inflammatory response; reduced chance of infection; reduced resorption; reduced extrusion rates	Limited donor site; can be poor quality such as septal fractures
Auricular cartilage (autologous)	Easily accessible; pliable cartilage; minimal donor site morbidity; minimal inflammatory response; reduced chance of infection; reduced resorption; reduced extrusion rates	Post auricular incision; slightly longer procedure time
Costal cartilage (autologous)	Ample quantity; minimal inflammatory response; reduced chance of infection; reduced resorption; reduced extrusion rates	Frequently associated with warping; significant risks of donor site morbidity including pneumothorax, higher post-operative pain and chest wall scarring; longer procedure time or two surgical teams required
Costal cartilage (cadaveric)	Ample quantity; no additional procedure for patient	Frequently associated with warping; ossification in the elderly; significant risks of donor site morbidity including pneumothorax, higher post-operative pain and chest wall scarring; increased inflammatory response; increased chance of infection; increased resorption; increased extrusion rates

The results of our study suggest overall high patient satisfaction at the donor site when conchal cartilage is harvested in septorhinoplasty surgery. This is in spite of outcomes affirming previously published objective data about post-operative changes in shape and symmetry of the donor ear [5]. Indeed, the results imply that although patients notice this cosmetic difference at the donor site, there is a minimal impact on their overall confidence and quality of life.

PROMs investigating donor site outcomes traditionally focus on autologous costal cartilage [12]. The majority of existing literature on patient satisfaction in the context of reconstructive surgery involving the ear and autologous cartilage centres on microtia repair, where the pinna is the reconstructive target rather than the donor site [13]. However, we noted research by Ho et al., who studied donor site PROMs in physical domains where both rib and ear cartilage was harvested for septorhinoplasty [14]. This group demonstrated that post-operative donor-site ear pain was perceived as moderate intensity, though pain scores diminished over time. There was no significant difference found in donor pain perception between the rib and ear. Indeed, this was replicated among the physical domains in our study, where pain perception had the lowest mode for a “never” response (66%, n=8).

Nevertheless, our results suggest high patient satisfaction in the other physical domains. Notably, the majority of patients (n=10) reported no post-operative issues with hearing, despite potential alteration in external ear anatomy. Amin et al. published an objective assessment of the relationship between pinna morphology and hearing, using rubber-composite models coupled with a sound processor, generating decreased sound intensity detection with wedge resection [15]. However, this was significantly attenuated where defects were closed, suggesting that maintenance of pinna continuity following cartilage harvest can limit development of post-operative hearing issues.

Although the PROMs included herein provide useful preliminary data, there were several limitations of this study. The overall cohort of patients incorporated was low, with a lack of homogeneity in follow-up duration. Inclusion of data from a greater number of surgeons may alter the overall conclusions through a greater cohort of patients, although this may have an impact on uniformity due to variations in surgical technique. In addition, outcomes were dependent on inherently subjective data, and using adjunctive tools to obtain objective measurements would refine our study. Data collection was performed retrospectively, and additional information about the temporal changes in patient perception at each stage of follow-up could be obtained using a prospective approach. Furthermore, a non-validated PROM tool was utilised for data collection, and results are thus subject to measurement and analysis errors. An optimal design for future research would be to implement the use of a validated data collection tool, such that conclusions can be made with a greater degree of confidence. Including data on patients who have undergone cartilage harvest from the ribs would allow for comparative analysis of techniques. Analysis of the relationship between septorhinoplasty outcomes and patient perception of the auricular donor site would also be an area for further research.

## Conclusions

The results of this pilot study suggest high levels of patient satisfaction, with minimal physical and psychological donor site sequelae following auricular cartilage harvest in septorhinoplasty. Subsequent studies should involve the use of validated questionnaires to provide further data for analysis. The inclusion of a PROM database within the literature would assist surgeons during the pre-operative/planning process.
